# Babies Living Safe & Smokefree: randomized controlled trial of a multilevel multimodal behavioral intervention to reduce low-income children’s tobacco smoke exposure

**DOI:** 10.1186/s12889-017-4145-7

**Published:** 2017-03-14

**Authors:** Bradley N. Collins, Stephen J. Lepore

**Affiliations:** Department of Social and Behavioral Sciences, College of Public Health, Temple University, 1301 Cecil B. Moore Ave, Ritter Annex, 9th Floor, Philadelphia, PA 19122 USA

**Keywords:** Secondhand tobacco smoke, Randomized controlled trial, WIC, Prevention, Smoking cessation, Community health services, Multilevel

## Abstract

**Background:**

Addressing children’s tobacco smoke exposure (TSE) remains a public health priority. However, there is low uptake and ineffectiveness of treatment, particularly in low-income populations that face numerous challenges to smoking behavior change. A multilevel intervention combining system-level health messaging and advice about TSE delivered at community clinics that disseminate the Special Supplemental Nutrition Program for Women, Infants and Children (WIC), combined with nicotine replacement and intensive multimodal, individual-level behavioral intervention may improve TSE control efforts in such high-risk populations.

**Methods/Design:**

This trial uses a randomized two-group design with three measurement points: baseline, 3-month and 12-month follow-up. The primary outcome is bioverified child TSE; the secondary outcome is bioverified maternal quit status. Smoking mothers of children less than 6 years old are recruited from WIC clinics. All participants receive WIC system-level intervention based on the “Ask, Advise, Refer (AAR)” best practices guidelines for pediatrics clinics. It includes training all WIC staff about the importance of maternal tobacco control; and detailing clinics with AAR intervention prompts in routine work flow to remind WIC nutrition counselors to ask all mothers about child TSE, advise about TSE harms and benefits of protection, and refer smokers to cessation services. After receiving the system intervention, mothers are randomized to receive 3 months of additional treatment or an attention control intervention: (1) The multimodal behavioral intervention (MBI) treatment includes telephone counseling sessions about child TSE reduction and smoking cessation, provision of nicotine replacement therapy, a mobile app to support cessation efforts, and multimedia text messages about TSE and smoking cessation; (2) The attention control intervention offers equivalent contact as the MBI and includes nutrition-focused telephone counseling, mobile app, and multimedia text messages about improving nutrition. The control condition also receives a referral to the state smoking cessation quitline.

**Discussion:**

This study tests an innovative community-based, multilevel and integrated multimodal approach to reducing child TSE in a vulnerable, low-income population. The approach is sustainable and has potential for wide reach because WIC can integrate the tobacco intervention prompts into routine workflow and refer smokers to free evidence-based behavioral counseling interventions, such as state quitlines.

**Trial registration:**

Clinicaltrials.gov NCT02602288. Registered 9 November 2015.

## Background

Children’s tobacco smoke exposure is a leading cause of disease in children and is linked to behavioral problems. [1–3] Babies and preschoolers are at particular risk for tobacco smoke exposure (TSE) because of their dependence on others to avoid exposure. Residential smoking is the main source of child TSE, with maternal smoking having a primary effect on young children [4]. Therefore, addressing child TSE and maternal smoking remains a public health priority [5]. Because TSE is very high among impoverished children [2, 6], and poverty is linked to higher smoking rates among women of childbearing age [7, 8], effective TSE intervention targeting low-income maternal smokers is needed. Evidence-based treatments are available, but uptake of these treatments remains low in impoverished populations [9]. Community health systems serving low-income communities, such as the Special Supplemental Nutrition Program for Women, Infants and Children (WIC), could facilitate intervention uptake in this high-risk population.

It is well established that smoking is influenced by multiple factors [2] including physical addiction, learned behaviors and social factors, that interact with one another and maintain their influence across both temporal and physical contexts [10, 11]. However, most efforts to address maternal smoking in community settings typically use just a “single-level” approach [12] that attempts to address factors at either the individual, social, or environmental level of influence. Such approaches have limited effectiveness in changing smoking behavior [13]. Therefore, testing innovative multimodal, multilevel TSE interventions could advance the field [14, 15]. Such approaches could address a wider array of factors that maintain smoking behavior and increase the odds of quitting relative to any single-level intervention [16]. Multilevel approaches could also boost intervention dosage through multiple message channels across levels of influence, thereby increasing the potency of intervention. While different sources of messaging may differentially influence knowledge, attitudes and motivation to change [17], systematic multilevel messaging could produce interactive effective across levels of influence to augment motivation and behavior change [18]. However, multilevel approaches are underutilized [19], particularly when addressing maternal smoking and child TSE. Underutilization may be due to resource and logistics challenges in community clinic settings – constraints this trial attempts to overcome.

The Babies Living Safe and Smokefree (BLiSS) program addresses the current limitations to implementing smoking interventions in community settings, such as WIC clinics, by using multiple message sources and repeated doses of intervention components across integrated treatment modalities. Informed by our successful multilevel intervention in pediatrics clinics [20–22], the impact potential of this model is high as it addresses many determinants of maternal smoking and child TSE. First, we implement a WIC system-level “Ask, Advise, Refer (AAR)” intervention that is modeled after the American Academy of Pediatrics best practices guidelines for child TSE/tobacco control in clinic [23]. Messaging at this level of intervention is designed to increase parents’ awareness about TSE harms and the benefits of TSE protections, increase parents’ motivation to change smoking behavior, and link smokers to more evidence-based treatment (e.g., quitline). Second, participants receive behavioral counseling with a proactive quitline to guide adoption and maintenance of TSE protective behaviors, self-regulation and coping skills, while providing social support for modifying smoking behavior leading to a quit attempt. Third, a mobile app, text messaging and texted educational video clips are integrated with counseling messaging to increase treatment support, engagement and dosage. Finally, participants receive eight weeks of NRT to address the physiological level of nicotine addiction.

This trial evaluates the efficacy of the BLiSS model in a sample of urban-dwelling, predominantly low-income African American maternal smokers. Targeting this population with an intervention that exceeds the intensity of standard practice is warranted because this population bears greater tobacco-related morbidity and mortality risk [2, 5] and greater challenges to changing smoking behavior compared to the general population [7]. The BLiSS intervention is framed by the Behavioral Ecological Model [15], which logically integrates biological, social and ecological concepts with principles of behavior change and posits that smoking interventions should target multiple determinants of smoking. Thus, our multilevel approach capitalizes on contingencies of reinforcement for protecting children from TSE that function reciprocally across levels of influence, including (a) a clinic system-level (e.g., meta-contingencies that promote AAR best practices and a culture within WIC clinics that encourage TSE protection norms); the interpersonal level (e.g., counselor provision of social support and guidance with problem solving during quitline sessions); and the intrapersonal level (e.g., mobile app experience that extends WIC and quitline counselor advice about TSE reduction). The BLiSS intervention model represents an innovative approach in WIC clinics that uses evidence-based clinic and multimodal behavioral treatment elements informed by the literature, our previous research and theory [15, 20, 21, 24–26].

### Aims and hypotheses

Aim 1: Test the hypothesis that a program integrating a WIC system-level “AAR” intervention with an intensive multimodal behavioral intervention (AAR + MBI) will be more effective in reducing child TSE (primary outcome) than a WIC system AAR plus an attention control intervention (AAR + Control). *Hypothesis 1:* Compared with AAR + Control children, those in the AAR + MBI condition will have significantly greater reductions in parent-reported daily TSE and child urine cotinine from baseline to 3- and 12-month follow-up.

Aim 2: Test the hypothesis that the multilevel AAR + MBI will be more effective at increasing maternal quit rates (secondary outcome) than AAR + Control. *Hypothesis 2:* Compared with the control group, mothers in AAR + MBI will have a significantly greater bioverified, 7-day point prevalence quit rate at 3- and 12-month follow-up.

Aim 3: Test hypotheses that changes in theoretically important variables will mediate the effects of AAR + MBI on child TSE and parent smoking outcomes. *Hypothesis 3*: Compared with parents in the AAR + Control condition, parents in the AAR + MBI condition will evidence greater increases in TSE protective behaviors, social support, urge management coping skills, and self-efficacy related to protecting child from TSE and quitting smoking from baseline to 3-month follow-up. In turn, these changes in mediator variables will account for between-group differences in TSE and cessation outcomes at month 12 (e.g., change in child cotinine from baseline to 12-month follow-up).

Aim 4: Explore factors that may influence outcomes and moderate intervention effects, including presence of other smokers at home, nicotine dependence, depressive symptoms, weight concerns, and intervention dosage as measured by quitline, NRT, and app usage.

## Methods and Design

### Overview

This study uses a two-group (experimental vs. attention control), double blind randomized controlled design with three measurement points including pre-treatment baseline, 3-month (end of treatment) and 12-month follow-up. The target population is smoking mothers enrolled in Philadelphia, Pennsylvania’s WIC program – a community-based health system that serves over 70,000 low-income families annually. The primary outcome is bioverified child TSE and the secondary outcome is bioverified maternal smoking abstinence. The study design is guided by CONSORT criteria [27] and is approved by Temple University’s Institutional Review Board (protocol number 23188). In the clinics, WIC nutrition counselors will be trained to implement the Ask, Advise, Refer (AAR) [23] intervention for addressing child TSE and parental smoking. All smokers, including those who do not wish to be referred to the BLiSS trial, will receive the state quitline number, which also helps them to access NRT. Eligible consented mothers will complete baseline assessment and get randomized to receive one of the two interventions: (a) the multimodal behavioral intervention (AAR + MBI) which will include telephone-based quitline counseling that focuses on reducing child TSE and maternal smoking cessation, an integrated mobile app linked to a web-enabled counselor portal to facilitate self-monitoring, text messages and educational video clips related to smoking, and NRT; or (b) the attention control (AAR + Control) intervention that includes similar contact time AAR + MBI with telephone-based nutrition education, separate mobile app and text-message delivered educational video clips that focus on improving family nutrition.

### Participants

**Fig. 1:**
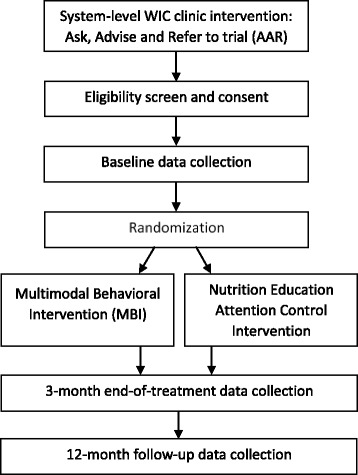
Flowchart of trial recruitment, assessment and intervention

The trial’s sample will be recruited in partnering WIC clinics. WIC mothers who have received the WIC clinic AAR intervention and are referred to the trial will be eligible if they are English-speaking, at least 18 years old, report smoking, own a smartphone, and report that their child under the age of 6-years old is exposed to tobacco smoke in their home. For mothers with multiple children under the age of 6, we will select the youngest child not in diapers or the oldest in diapers if all children are still in diapers. Exclusion criteria include pregnancy and presenting issues that can interfere with the ability to provide informed consent or follow study procedures, such as psychosis, inadequate health literacy, or (non-nicotine) drug dependence. Figure 1 shows participant flow through clinic intervention, enrollment, intervention, and data collection.

### Procedures

Prior to initiating participant recruitment, we will conduct a formative analysis of current tobacco intervention practices among professional and paraprofessional staff who work in Philadelphia WIC clinics. This preliminary research will facilitate the translation and implementation of AAR best practices into routine WIC clinic operations and client flow that will minimize both staff and participant burden. We will then provide group training among all staff that highlights the child TSE problem and its consequences, challenges maternal smokers have in quitting smoking, and the pivotal contributing role of community health workers in tobacco control efforts. In addition, WIC nutrition counselors will receive more intensive training in the specific steps to implementing the Ask, Advice, Refer intervention.

After WIC staff refer smoking mothers with exposed children to the trial, trained research assistants will consent eligible participants and collect self-report data at baseline. Data will be collected via structured computer assisted telephone interviewing. This method reduces literacy barriers, minimizes participant burden, and maximizes data reliability. After baseline, participants are randomized to condition. A permuted blocks design was used to randomize participants stratified by clinic site and presence of other smokers in the home living with the participant (yes/no). The randomization schedule was seeded using values obtained from random.org and the project biostatistician provided the allocations to the data collection team in sealed opaque security envelopes. To preserve condition masking, participants will be randomized after baseline data collection and immediately prior to a treatment orientation home visit. During the orientation home visit, participants receive a binder with intervention materials that are relevant to their assigned intervention condition. Research staff also download a mobile app specific to condition on participants’ smartphones and show an app video tutorial, the link to which is left with the participant. The child’s urine cotinine sample is also collected during the home visit.

Research staff blind to treatment assignment will conduct 3-month end-of-treatment and 12-month follow-up via structured telephone interviews. After each telephone assessment, another home visit is scheduled to obtain child urine cotinine samples and participant saliva to bioverify child TSE level and participant smoking status, respectively. In addition to a saliva sample, participants provide an expired CO sample given the potential that participants may be using NRT. Various procedures are used to reduce attrition, including reminder postcards and text messages, flexible scheduling, and financial retention incentives for completing assessments.

### Interventions

#### WIC system intervention (both groups)

Specific procedures for delivering the WIC system intervention includes the following steps: At each clinic visit, mothers complete a 3-item smoking and TSE survey included in their pre-appointment paperwork. A nutrition counselor reviews the survey and offers all mothers with exposed children advice about dangers of TSE and benefits of reducing exposure. Mothers also receive a pamphlet reiterating counselors’ TSE health messaging (e.g., “There is no safe level of TSE; ask your WIC counselor about the BLiSS program and other smoking treatment services”). Nutrition counselors discuss the BLiSS program and mark on the survey whether smoking mothers want a referral to the trial. Project staff proactively call these mothers for eligibility screening.

We facilitate assimilation of the protocol into routine WIC clinic work flow through adoption, implementation, and maintenance phases. During the adoption phase, research staff will provide system-wide training for all professional and paraprofessional WIC staff about maternal smoking and child TSE. This training also highlights the relevance of creating a pro-TSE-reduction culture and social norms within the WIC organization and how such norms may impact tobacco control efforts in the community. During implementation, project staff will meet with WIC counselors at each clinic sequentially to discuss the BLiSS program in the context of improving the quality of WIC clients’ care. WIC counselor input guides procedures that embed the AAR protocol in existing workflow to minimize staff and client burden. BLiSS staff will model and role play execution of the AAR protocol and troubleshoot barriers to AAR delivery to maximize adherence. Similar to our previous trial [20], implementation also involves academic detailing, which includes hanging posters and placing pamphlets in WIC clinics that reflect norms supporting child TSE reduction efforts, provide consistent health messaging across multiple modes of intervention, and prompt nutrition counselors to advise and refer smoking mothers to the trial. During the maintenance phase, project staff will provide AAR fidelity monitoring reports to the WIC management team and nutrition counselors that include number of referrals received and referred mothers’ reports about the TSE advice and written materials they received at WIC.

### Attention control intervention: nutrition education (AAR + Control)

Trained health counselors deliver the Control intervention. Training, in-home intervention orientation, and multimodal behavioral intervention procedures parallel procedures described in the MBI arm below. However, the content of the Control behavioral intervention centers on improving nutrition on a budget, with an emphasis on reducing sugar intake and increasing fruit and vegetable consumption. The purpose of the Control group is to provide equivalent attention and contact between the two conditions while providing distinctly different behavioral intervention content. At the orientation, BLiSS staff offers participants the state quitline number including information that the quitline delivers free NRT. Staff also provide the written tool kit developed by Sesame Street Workshop, *Food for Thoughts: Eating Well on a Budget* [28], that includes nutrition guidelines, recipe cards, and colorful books with activity suggestions as well as videos about nutrition on a budget and fruit and vegetable consumption. Additionally, staff use food models to demonstrate recommended serving sizes and download the Fooducate mobile app (with video tutorial) to participant smartphones. This app scans barcodes on foods to provide real time nutrition information. Staff also provide participants with the number of the state quitline and are informed they can receive smoking cessation counseling and free NRT by calling the number. Finally, during the weeks between the orientation and 3-month end-of-treatment phone assessment, participants will receive up to 5 telephone nutrition education calls, 10 nutrition education video clips via text messaging, and inter-session text message follow-ups and appointment reminders.

### Experimental multimodal behavioral intervention (AAR + MBI)

Prior to implementing the experimental MBI, counselors will complete a 5-day Certified Tobacco Treatment Specialists training accredited by the Association for Treatment of Tobacco Use and Dependence. An additional 30 h of training by Collins (PI) will expand counselors’ skills including (a) how to guide TSE protection efforts as part of a cognitive behavioral therapy (CBT)-framed strategy to shape parents’ behavior toward a cessation attempt; (b) how to facilitate TSE protective behaviors and smoking behavior change in the context of challenges faced by low-income parents who smoke; and (c) how to drive a counseling process that integrates support and skills training with advice that facilitates participants’ mobile app usage to extend individualized treatment beyond phone sessions. Training in CBT strategies allows for tailoring counseling to participants based on identified barriers (e.g., other smokers in the home) and catalysts (e.g., concerns for child health) of behavior change. After intensive competency-based training, counselors will refine skills in ongoing weekly supervision that includes review of fidelity monitoring feedback, discussion of current cases, and role-playing of effective session interaction.

Intervention components were informed by the current literature and our two previous smoking trials with low-income parents [20, 21, 26]. Components include integrated health messaging that parallels WIC counselor advice as well as support and guidance with skills training and problem solving that is delivered via multiple treatment modes: (a) a project quitline that provides up to 5 telephone counseling sessions during the weeks between orientation and 3-month end-of-treatment phone assessment; (b) a research-ready BLiSS mobile app; (c) print materials for the participant and their family; (d) inter-session text follow-up, reminders and support as well as scheduled educational video clips that highlight telephone session and written materials content; (d) 8 weeks of NRT and instructional support.

At treatment orientation, staff will provide a printed participant guide to the intervention with treatment schedule and content that parallels upcoming counseling, and a printed family guide to BLiSS that suggests ways to navigate family-level barriers to child TSE adoption and smokefree home adoption. Counselors review materials such as behavioral contracts for a smokefree home, “no smoking” signs, information about the importance of NRT, and advice about proper NRT use. Counselors also download the BLiSS app to participants’ smartphones, oversee participants’ viewing of our app tutorial video and review a printed app instruction pamphlet.

#### Telephone counseling “quitline”

The quitline is the integral component within the MBI. Phone counseling is an emerging standard of care that is efficacious, acceptable and can reach disadvantaged populations [29–31]. NRT can also be distributed by quitlines [32]. The timing and frequency of telephone sessions is guided by best practices, including multiple proactive calling and at least 2–3 completed calls [29, 33, 34]. We plan to offer 5 proactive calls with flexible scheduling to promote TSE protections, to shape TSE reduction achievements toward smoking cessation, and provide the extra support for skills training needed in our target population. We will also send frequent reminder messages via text and voicemail. The counseling schedule and content mirrors strategies used in our previous trials [20, 26] and common evidence-based practices implemented in state and national quitline counseling models [32]. Counseling content is also guided by recent evidence and approaches that use motivational components to promote family-level support for and adoption of TSE protective behaviors among household members by capitalizing on parents’ desire to protect young children from TSE [35, 36]. Key counseling components in BLiSS include: (a) increasing motivation for smoking behavior change with collaborative, individualized treatment plans, support with goal setting, and guidance for building social support; (b) addressing addiction with education about NRT and proper use; (c) improving skills (e.g., self-monitoring) to identify smoking “triggers” and to manage smoking urges with compensatory CBT strategies; (d) improving cessation self-efficacy; and finally (e) coaching and feedback about participants’ BLiSS mobile app usage facilitated by a web-linked counselor portal (dashboard) to encourage real-time self-monitoring and coping skills training.

#### BLiSS mobile app

Quitline standard practice [33] encourages the provision of smoking cessation mobile apps. However, unlike the BLiSS app, few smoking cessation apps adhere to practice guidelines or link to evidence based treatment [37]. The BLiSS mobile app is a modified version of the National Cancer Institute's former Quitpal mobile app [38] that participants can use on iPhone and Android platforms. The app includes features that facilitate real-time self-monitoring of cigarettes smoked, child TSE and trigger/urge associations. Participants also enter their quit date, track health progress and can view information about health improvements related to TSE protection and cessation and a goal progress summary. The app also records times and triggers participants enter each day, and on subsequent days, pushes urge management strategies and reminders (e.g., NRT use) at those times to encourage motivation and provide supportive guidance and positive reinforcement of their efforts.

An innovative feature of the BLiSS app is the web-linked counselor portal with a dashboard that displays participant app usage. The dashboard guides supportive feedback from health counselors around self-monitoring without adding participant burden, thereby enabling “supportive accountability” which theory and empirical evidence suggest could facilitate more effective mHealth use [39]. Counselors will discuss and troubleshoot participants’ app usage to and provide positive reinforcement for their tracking efforts with the app. Such exchanges will increase intersession intervention dosage around individualized skills training.

#### Nicotine replacement therapy starter and instructions

NRT products will be provided to participants (specifically, nicotine gum, lozenge or patch). NRT is FDA approved for smoking cessation interventions and available over the counter. Following best-practice guidelines and practices implemented by state quitlines with low-income clients [32, 40], we will mail participants up to 8-weeks of free NRT prior to their quit day. Project quitline counselors will give advice on NRT use based on guidelines including benefits and harms, safe use and disposal.

#### Text messaging and educational video clips

To supplement written materials provided during the orientation and counseling messaging throughout treatment, we will also provide frequent text messaging. One purpose of text messaging is to remind and encourage participants’ adherence to scheduled telephone sessions. Another purposed is to extend messaging around key components of the behavioral intervention. This is achieved with brief, post session summary of primary individualized content and advice to emphasize intersession goals and skills training “homework.” Health messaging is also extended with 10 scheduled text messages with attached brief animated videos highlighting essential components of the intervention. Examples of video topics include: the importance of goal setting; setting up smokefree zones as a step toward a smokefree home; overview of coping skills to manage smoking urges; managing weight and worry; relapse prevention. In a further attempt to integrate health messaging and skills training across modes of intervention, we use avatars of our telephone counselors in our written materials and video clips. Text messages also are used to prompt adherence to using the mobile app.

### Measures

#### Outcome variables

Child TSE is the primary outcome and is assessed using two methods that yield continuous variables. First, via validated timeline follow-back assessment [41], parents will report the number of cigarettes to which the child is exposed each day during the 7 days prior to all assessment periods. Child cotinine will be collected in urine samples at home visits following each phone assessment. The secondary outcome is parent smoking status (1 = quit; 0 = not quit). During phone interviews at 3- and 12-month follow-ups, staff will obtain parent self-reported 7-day point prevalence abstinence and prolonged abstinence [42]. Self-reported point prevalence abstinence will be bioverified with salivary cotinine (Nicalert™) and expired CO at home visits following the telephone assessments.

#### Mediators

Four variables will be assessed as potential mediators: (a) Parental-reported exposure protection (PREP) behaviors are measured with self-report items used in our previous research and reflected in a recent meta-analysis. We operationalize PREP behavior in two measures: (1) families’ current home smoking policy (0 = no restrictions to 4 = total indoor ban); and (2) the sum of 10 PREP behaviors (e.g., move child away from others’ smoking). (b) Support for TSE protection and smoking cessation will be measured using a modified short form of the Partner Interaction Questionnaire. The modified measure, used in our previous multilevel trial [20], assesses perceived intervention staff support to participants for promoting TSE reduction and cessation (α = .91). (c) Self efficacy of smoking behavior change will be measured using the Smoking Cessation Self-Efficacy Scale (α = .90) and a parallel form (α = .90) we developed [20] to assess self-efficacy in reducing child TSE. (d) Urge management and coping will be assessed using the Urge Management Coping Skills measure [20] based on cognitive and behavioral coping strategies identified by O’Connell (α = .87).

#### Covariates and moderators

Four variables will be assessed as potential control variables and moderators of intervention effects: (a) Nicotine dependence will be measured with the reliable and validated Fagerström Test for Cigarette Dependence [43]. (b) Depressive symptoms will be measured with the Center for Epidemiological Studies Depression Scale, which are validated, reliable and useful in smoking studies [44]. (c) Weight concerns will be measured using a 6-item validated scale that measures general and smoking-specific weight concerns [45]. (d) Other smokers in mothers’ homes will be assessed using a standard, content valid item. Additional demographic and smoking history variables will be measured and assessed for possible association with outcomes.

#### Process measures and treatment fidelity

Intervention processes will be assessed in both conditions with multiple methods and at each time point and intervention level: WIC clinic AAR implementation (e.g., participant reports of advice received; WIC counselor report of attitudes and practices), telephone counseling (e.g., counselor reports of attendance, duration, participant engagement), NRT utilization (e.g., participant-reported compliance), and mobile app usage (e.g., pushed data reflecting frequency of launching and using specific features).

### Analytic plan

The predictor is intervention group (AAR + MBI vs. AAR + Control) and the two endpoints include child exposure (continuous primary outcome) and parent cessation (dichotomous secondary outcome). Primary analyses will use an intention-to-treat approach and missing data will be addressed using multiple imputation. In sensitivity analyses, we will perform complete case analyses and investigate the impact of data that is potentially missing not at random. We will compare child cotinine change scores between intervention arms using multilevel random-effects linear regressions in which we include randomization arm as a fixed effect and WIC clinic as a random intercept to account for potential clustering within clinic. Potential confounders (e.g., nicotine dependence) will be added as covariates as necessary. We will use multiple logistic regressions with random intercepts for clinic to compare bioverified self-reported quit status (yes/no) between randomization arms at 3 and 12 months. To investigate mediation pathways, we will use a structural equation approach with change in cotinine levels and parent quit status as the outcomes. We will ensure that the intervention is associated with the potential mediators (e.g., self-efficacy) and that the mediators are associated with the outcomes. We will also use cluster-adjusted bootstrap standard errors for assessment of the mediated effect in which the variables are measured in a temporal ordering consistent with a causal pathway. For moderation analyses, we will use random-effects multiple regression modeling the change in cotinine levels and parent quit status, using confounding variables as necessary.

#### Power analysis

We chose our sample size so that we would have at least 85% power to detect between-group differences in the primary outcomes (3- and 12-month child cotinine). Using an estimate of 20% 1-year follow-up attrition based on the KiSS trial, we expect to have 298 participants at 12 months (372*80% retention = 298, or 149 per group). With 149 participants per group, we will have 85.7% power to detect an effect of 0.23 in the log cotinine change scores between randomization arms.

## Discussion

This project will address current limitations to tackling maternal smoking and child TSE in low-income populations by testing the efficacy of an appropriately comprehensive, community-based multilevel intervention in a high-risk population known to face numerous challenges to smoking behavior change. NIH has asserted that testing such interventions in high-risk populations is a public health priority [46]. If efficacious, the Babies Living Safe and Smokefree (BLiSS) intervention model offers a tobacco control prototype for the WIC system and informs state quitlines that serve high-risk families. Implementing BLiSS throughout established community health clinics (e.g., WIC) and state quitlines holds substantial potential to reach disparate, high-risk groups and could lead to profound public health benefits by reducing health and cost burdens associated with tobacco use and exposure in populations presenting excess tobacco morbidity and mortality.

A clinic-level intervention such as BLiSS enhances the quality of care clients receive in WIC, and it represents a pragmatic approach to community-based tobacco control that mirrors the Ask, Advise, Refer best practices in pediatrics clinics. However, because research has established that brief standalone interventions are not highly efficacious in smoking populations known to face greater challenges to cessation, there is a need for an intensive multilevel, multimodal intervention such as BLiSS. The BLiSS intervention model is the most feasible and sustainable multilevel intervention concept to date because it can be deployed where the largest smoking burden exists with minimal burden on staff. The integrated multilevel model may effectively link low-income smokers to intensive multimodal behavioral intervention that augments WIC counselor advice, promotes necessary support and skills training via telephone counseling and integrated mobile app, and provides NRT with usage guidance. Unlike current quitlines that may avail clients to app and social media intervention platforms, our multimodal components are explicitly integrated with the purpose of reiterating consistent health messaging, advice and support across platforms and extending counseling to real-time support and skills training through guided mobile app usage facilitated by the counselor dashboard that displays participant app usage. Other contributions of this research include the collection of usage data from the BLiSS app. These process data will guide future directions for integrating contemporary mHealth technology with behavioral counseling for multimodal tobacco interventions.

## Abbreviations

AAR: Ask, Advise, Refer
BLiSS: Babies living safe and smokefree
MBI: multimodal behavioral intervention
NRT: nicotine replacement therapy
PI: principal investigator
TSE: tobacco smoke exposure
WIC: women infants and children clinics

## References

[1] World Health Organization (2009). WHO report on the global tobacco epidemic, 2009: implementing smoke-free environments.

[2] U.S. Department of Health and Human Services. The Health Consequences of Involuntary Exposure to Tobacco Smoke: A Report of the Surgeon General. Atlanta, GA; 2006. https://www.ncbi.nlm.nih.gov/books/NBK44324/. Accessed 2 Jan 2017.

[3] Twardella D, Bolte G, Fromme H, Wildner M, von Kries R, GME Study Group (2010). Exposure to secondhand tobacco smoke and child behaviour - results from a cross-sectional study among preschool children in Bavaria. Acta Paediatr.

[4] Stiby AI, Macleod J, Hickman M, Yip VL, Timpson NJ, Munafò MR (2013). Association of maternal smoking with child cotinine levels. Nicotine Tob Res.

[5] U.S. Department of Health and Human Services. Maternal, infant, and child health. 2020 topics and objectives. 2014;15 SRC-. https://www.healthypeople.gov/2020/topics-objectives/topic/maternal-infant-and-child-health?topicid=26. Accessed 2 Jan 2017.

[6] Centers for Disease Control and Prevention (CDC) (2009). Cigarette smoking among adults and trends in smoking cessation. Morb Mortal Wkly Rep.

[7] Tong VT, Jones JR, Dietz PM, D’Angelo D, Bombard JM, Centers for Disease Control and Prevention (CDC) (2009). Trends in smoking before, during, and after pregnancy - Pregnancy Risk Assessment Monitoring System (PRAMS), United States, 31 sites, 2000-2005. MMWR Surveill Summ.

[8] Melvin C, Gaffney C (2004). Treating nicotine use and dependence of pregnant and parenting smokers: an update. Nicotine Tob Res.

[9] Hiscock R, Bauld L, Amos A, Fidler JA, Munafò M (2012). Socioeconomic status and smoking: a review. Ann N Y Acad Sci..

[10] Collins BN, Nair US, Komaroff E (2011). Smoking cue reactivity across massed extinction trials: negative affect and gender effects. Addict Behav.

[11] Collins BN, Brandon TH (2002). Effects of extinction context and retrieval cues on alcohol cue reactivity among nonalcoholic drinkers. J Consult Clin Psychol.

[12] Anderson NB (1998). Levels of analysis in health science. A framework for integrating sociobehavioral and biomedical research. Ann N Y Acad Sci.

[13] Lando HA, Valanis BG, Lichtenstein E (2001). Promoting smoking abstinence in pregnant and postpartum patients: a comparison of 2 approaches. Am J Manag Care.

[14] NIH Science of Behavior Change - Meeting Report.; 2009. https://commonfund.nih.gov/behaviorchange/meetings/sobc061509/index. Accessed 2 Jan 2017.

[15] Hovell MF, Hughes SC (2009). The behavioral ecology of secondhand smoke exposure: A pathway to complete tobacco control. Nicotine Tob Res.

[16] U. S. Department of Health and Human Services. Clinical Practice Guideline. Treating Tobacco Use and Dependence: 2008 Update. 2008;2009 SRC. https://bphc.hrsa.gov/buckets/treatingtobacco.pdf. Accessed 4 Jan 2017.

[17] Lavery A, Nair US, Bass S, Collins BN (2016). The Influence of Health Messaging Source and Frequency on Maternal Smoking and Child Exposure among Low-Income Mothers. J Commun Healthc Strateg Media Engagem Glob Heal.

[18] Kottke TE, Solberg LI, Brekke ML, Maxwell P (1988). Smoking cessation strategies and evaluation. J Am Coll Cardiol.

[19] Reus VI, Smith BJ (2008). Multimodal techniques for smoking cessation: a review of their efficacy and utilisation and clinical practice guidelines. Int J Clin Pract.

[20] Lepore SJ, Winickoff JP, Moughan B (2013). Kids Safe and Smokefree (KiSS): a randomized controlled trial of a multilevel intervention to reduce secondhand tobacco smoke exposure in children. BMC Public Health.

[21] Collins BN, Nair US, Godfrey M (2015). Kids Safe and Smokefree: A multilevel trial to protect children from tobacco smoke and promote cessation among low-income parents. Society for Research in Nicotine and Tobacco Europe.

[22] Collins B, Nair U, Godfrey M, Hunt-Johnson A, Lepore S. Linking pediatric system- and individual-level tobacco interventions: A multilevel approach to addressing parental smoking in a vulnerable population. In Symposium: "You Can’t Stop Smoking? New Therapeutic Approaches to Smoking Cessation. In: Association for Behavior and Cognitive Therapy. Vol. U. S. Association: Association for Behavioral and Cognitive Therapy; 2016.

[23] American Academy of Pediatrics. Counseling about smoking cessation. 2011;2011 SRC. http://www2.aap.org/richmondcenter/CounselingAboutSmokingCessation.html. Accessed 4 Jan 2017.

[24] Collins B, Lepore S, Winickoff JP, et al. Improving efforts to reduce vulnerable children’s exposure to tobacco smoke: Integrating health systems and individual interventions in a multilevel randomized trial. In: Vol Denver, CO; American Public Health Association; 2016.

[25] Bandura A (2004). Health promotion by social cognitive means. Health Educ Behav.

[26] Collins BN, Nair US, Hovell MF (2015). Reducing underserved children’s exposure to tobacco smoke: A randomized counseling trial with maternal smokers. Am J Prev Med.

[27] Schulz KF, Altman DG, Moher D, Consort Group (2010). CONSORT 2010 Statement: updated guidelines for reporting parallel group randomised trials (Chinese version). Zhong Xi Yi Jie He Xue Bao.

[28] Workshop S (2010). Food for thought: Eating well on a budget.

[29] Stead LF, Hartmann-Boyce J, Perera R, Lancaster T (2013). Telephone counselling for smoking cessation. Cochrane Database Syst Rev.

[30] Zhu SH, Anderson CM, Johnson CE, Tedeschi G, Roeseler A (2000). A centralised telephone service for tobacco cessation: the California experience. Tob Control.

[31] Vidrine D, Arduino R, Lazev A, Gritz E. A randomized trial of a proactive cellular telephone intervention for smokers living with HIV/AIDS. *Aids*. 2006. http://journals.lww.com/aidsonline/Abstract/2006/01090/A_randomized_trial_of_a_proactive_cellular.14.aspx. Accessed 2 Feb 2016. 10.1097/01.aids.0000198094.23691.58 16511419

[32] NAQC (2012). North American Quitline Consortium. Quitline Services offering models: A review of the evidence and recommendations for practice in times of limited resources.

[33] Anderson C (2016). Quitline Services: Current Practice and Evidence Base: NAQC Quality Improvement Initiative.

[34] McAfee T. Developing and Improving Toll-Free Tobacco Quit Line Services. A World Health Organization Manual. Geneva: WHO report; 2011. http://apps.who.int/iris/bitstream/10665/44738/1/9789241502481_eng.pdf. Accessed 5 Jan 2017.

[35] Kegler MC, Escoffery C, Bundy L, et al. Pilot study results from a brief intervention to create smokefree homes. J Environ Public Health. 2012. 10.1155/2012/951426 PMC3362929 22675374

[36] Stotts AL, Northrup TF, Schmitz JM (2013). Baby’s Breath II protocol development and design: A secondhand smoke exposure prevention program targeting infants discharged from a neonatal intensive care unit. Contemp Clin Trials.

[37] Abroms LC, Padmanabhan N, Thaweethai L, Phillips T (2011). iPhone apps for smoking cessation: a content analysis. Am J Prev Med.

[38] Killam B. User Centered Design Homepage. http://www.user-centereddesign.com/. http://www.user-centereddesign.com/. Accessed 5 Jan 2017.

[39] Mohr DC, Cuijpers P, Lehman K (2011). Supportive accountability: A model for providing human support to enhance adherence to eHealth interventions. J Med Internet Res.

[40] U.S. Department of Health and Human Services. Best Practices for Comprehensive Tobacco Control Programs—2014. Centers Dis Control Prev Natl Cent Chronic Dis Prev Heal Promot Off Smok Heal 2014. 2014. http://www.cdc.gov/tobacco/stateandcommunity/best_practices/index.htm?source=govdelivery.

[41] Brown RA, Burgess ES, Sales SD, Whiteley JA, Evans DM, Miller IW (1998). Reliability and validity of a smoking timeline follow-back interview. Psychol Addict Behav.

[42] Hughes JR, Keely JP, Niaura RS, Ossip-Klein DJ, Richmond RL, Swan GE (2003). Measures of abstinence in clinical trials: issues and recommendations. Nicotine Tob Res.

[43] Fagerstrom K (2012). Determinants of tobacco use and renaming the FTND to the Fagerstrom Test for Cigarette Dependence. Nicotine Tob Res Off J Soc Res Nicotine Tob.

[44] Andresen EM, Malmgren JA, Carter WB, Patrick DL (1994). Screening for depression in well older adults: evaluation of a short form of the CES-D (Center for Epidemiologic Studies Depression Scale). Am J Prev Med.

[45] Borrelli B, Mermelstein R (1998). The role of weight concern and self-efficacy in smoking cessation and weight gain among smokers in a clinic-based cessation program. Addict Behav.

[46] National Institutes of Health. Trans-NIH Integrated Health Strategies Working Group: Advancing the Science of Effective Behavioral Treatments in Primary Care. Bethesda; 2010. https://www.nimh.nih.gov/about/advisory-boards-and-groups/namhc/reports/directors-report-to-the-225th-national-advisory-mental-health-council-meeting-may-14-2010.shtml. Accessed 5 Jan 2017.

